# Selection/control concurrent optimization of BLDC motors for industrial robots

**DOI:** 10.1371/journal.pone.0289717

**Published:** 2023-08-16

**Authors:** Erick Axel Padilla-García, Héctor Cervantes-Culebro, Alejandro Rodriguez-Angeles, Carlos Alberto Cruz-Villar

**Affiliations:** 1 Academia de Ingeniería en Robótica, Universidad Politécnica de Atlacomulco, Atlacomulco, Estado de México, México; 2 Escuela de Ingeniería y Ciencias, Tecnologico de Monterrey, Atizapán de Zaragoza, Estado de México, México; 3 Departamento de Ingeniería Eléctrica, Sección de Mecatrónica, CINVESTAV-IPN, Gustavo A. Madero, Mexico City, México; Guangdong University of Technology, CHINA

## Abstract

This paper aims to concurrently select and control off-the-shelf BLDC motors of industrial robots by using a synergistic model-based approach. The BLDC motors are considered with trapezoidal back-emf, where the three-phase (a,b,c) dynamics of motors are modeled in a mechatronic powertrain model of the robot for the selection and control problem, defining it as a multi-objective dynamic optimization problem with static and dynamic constraints. Since the mechanical and electrical actuators’ parameters modify the robot’s performance, the selection process considers the actuators’ parameters, their control input, operational limits, and the mechanical output to the transmission of the robot joints. Then, three objective functions are to be minimized, the motor’s energy consumption, the tracking error, and the total weight of installed motors on the robot mechanism. The control parameterization approach via a cascade controller with PI controllers for actuators’ voltage and a PID controller for actuators’ torque is used to solve the multi-objective dynamic optimization problem. Based on simulations of the closed-loop system, a Pareto front is obtained to examine trade-offs among the objective functions before implementing any actuators in the existing robotic system. The proposed method is tested on an experimental platform to verify its effectiveness. The performance of an industrial robot with the actuators originally installed is compared with the results obtained by the synergic approach. The results of this comparison show that 10.85% of electrical power can be saved, and the trajectory tracking error improved up to 57.41% using the proposed methodology.

## Introduction

An industrial robot is a highly integrated system where mechanical, electronic, and information technology subsystems concur to render a mechatronic system, where the balance among subsystems impacts the robot’s overall performance. Several optimization-based methods have been proposed to find an optimal balance or a so-called synergy among subsystems.

Typically, optimal design methodologies address the mechanism and controller integration by assuming the actuator or actuators are given. In [1], a serial two-link high-speed arm is optimally designed to minimize the robot settling time. In contrast, the design parameters are actuator locations, feedback gains, and arm link geometry. The desired task plays an essential role for ultrahigh-performance robots and could be considered since the design stage along with kinematic, dynamic, and control parameters, [2]. Most industrial robots are embedded in well-structured environments. However, uncertainties such as manufacturing tolerances or environmental changes are always present, so a robust formulation for the structure/control optimal design of mechatronic systems is proposed in [3]. Recently, ontologies have been introduced to define a set of representational primitives modeling the relationships among two domains intersecting in a mechatronic system, mechanical domain, and feedback control domain, [4].

Industrial robot powertrain design has been performed employing optimization procedures. [5] use an optimization strategy to find the powertrain for new robot concepts when cost and lifetime are considered performance index. The trajectory generation task is included in the design strategy. Service robots should be fulfilled with a lightweight mechanism with desired kinematic performance and compliance. [6] propose an integrated design optimization approach where robot kinematics, dynamics, powertrain design, and strength analysis are considered. In such an approach, kinematic and structural dimensions, motors, and gearboxes are parameterized as design variables. Customized designs of serial manipulators, [7], are usually performed via a two-step optimization strategy. The first step determines the maximum load estimation at each robot joint. In contrast, the second step selects an appropriate motor-gear assembly for the joint, providing an appropriate weight estimation and evaluating the payload capacity of each joint.

The optimal mechanical design combined with dynamic control avoids a sub-optimal behavior when path-tracking controller parameters are disregarded in the power-train selection process, [8]. A concurrent multi-objective dynamic optimization method is proposed for optimal selection and control of Permanent Magnet Synchronous Motors (PMSM) driving an industrial parallel robot as in [9].

PMSM motors exhibit high precision and torque in industrial robots and applications. However, the combination of PMSM motors and powertrains is heavy and bulky, [10]. BLDC motors can replace PMSM motors since they are lighter to overcome this problem. In addition, BLDC motors have different control requirements and operating conditions, [11]. For instance, the BLDC rotor spinning induces a trapezoidal-shaped Back Electro-Motive Force (BEMF) instead of sinusoidal, where the magnet flux linkage varies with changes in the motor current, making it unsuitable for the sinusoidal Clark’s and Park’s transformation [12], as used in the PMSM models. Then, the non-sinusoidal a,b,c phase variable method is preferred to model the BLDC Motor dynamics, obtaining the dynamic voltages and currents of the 3-phase system, in contrast with PMSM models, where they are usually transformed into a stationary two-phase reference system [13]. However, the electrical control of BLDC motors requires alternating stator current to feed the motor coils every 60 degrees, which means that a switching current control method is needed for a specific commutation sequence to control the motors instead of the well-known Field Oriented Control (FOC) method.

Numerous studies have been conducted to minimize specific phenomena, such as torque ripple in BLDC motors [14]. For instance, in [15], an Adaptive PID controller to regulate the speed of a BLDC motor, which improves its capacity and reduces improper irregular generation. Optimal gain values are proposed to reduce torque ripples, and a hybrid method combining Harris Hawks Optimization (HHO) and Black Widow Optimization (BWO)-based Luo converter is used for optimization. The suggested regulation technique estimates EMF, stator current, torque, and speed. Another technique [16] proposes the Improved Jellyfish Search (ImpJS) technique is presented for minimizing torque ripple on a BLDC motor with a CUK converter. Crossover and mutation operators enhance the JS algorithm and improve the speed and torque control strategy. ImpJS also upgrades the controller operation by tuning the best gain parameter. The performance of the ImpJS system using MATLAB/Simulink is analyzed and then compared with an existing system. The Jaya Optimization Algorithm on the Xilinx platform to minimize the ripples in the motor’s torque is exposed in [17]. This control strategy is achieved by combining Xilinx and optimization algorithms. First, the dynamic mode of the BLDC motor is obtained and then electronically interacts with the motor using the Math lab/Simulink model while utilizing Xilinx tools for co-simulation. Energy consumption in industrial applications is another significant concern in the literature. The research ranges from maximizing solar energy extraction in electric vehicles using BLDC motors [18] and reducing energy consumption in industrial robots by selecting appropriate motors, drives, controllers, and techniques to minimize idle time [19].Regarding the optimal selection of motors and reducers, a method is presented in [20] that optimizes servo-axis component selection without iterative processes. It creates an electromechanical model that considers electrical and mechanical factors, with a size index for precise part selection. This method eliminates experimental characterization and allows for determining the optimal selection for the most general case. The proposed method allows users to confidently determine the feasibility of selecting commercial components. A similar approach is established in [21]; this paper aims to design and select servo-actuated systems concurrently for optimizing energy consumption. The method involves scaling rules that simplify the system’s characteristics into two key parameters: gearbox transmission ratio and continuous motor torque at stall. These scaling rules summarize the complex relationship between system parameters and energy consumption and are incorporated into the analytic formulation of overall energy consumption. The design problem can be cast as a constrained optimization problem with just two design variables using metamodels generated from data provided in datasheets. The entire process is automated and does not require any design iteration.

In this paper, we propose an optimization-based approach for the selection and control of off-the-shelf BLDC motors driving an industrial robotic system. Moreover, motor selection on the powertrain has several criteria that should be considered for BLDC motor types, such as torque per unit current, speed range, feedback devices, and parameter sensitivity. Regarding energy consumption, the electrical and mechanical power losses are considered from the three-phase dynamic voltages and currents. In a traditional transformed system, energy is obtained by steady-state conditions. Thus, in the optimization-based design, the sensitivity of the design variables impacts the outcome both in system performance and computational cost [22].

The energy consumption, the tracking error, and the motor weight at each robot joint are the performance indexes to be optimized, while the closed-loop system comprising the manipulator dynamics, the powertrain dynamics, and its controller states a set of differential equations as equality constraints. In our proposal, each off-the-shelf actuator is assigned an index number that identifies its electrical and mechanical parameters, thus heeding changes caused by fitting a given motor to the closed-loop dynamics. The controller’s gains for each motor, as well as its index number, are the set of selection/design independent variables for the resulting multi-objective mixed-integer nonlinear optimization problem, which is solved via the NSGA-II algorithm.

In the previous work, a robot with three degrees of freedom is modeled and simulated for a regulation task with PMSM [9]. This work proposes the optimal selection of BLDC motors and the controller gain tuning validated on an experimental platform using a five-DOF industrial robot. The implementation of dead-time compensation during the switching period of the commutation period is crucial in reducing the ripple effect in the current of a PWM three-phase inverter. Multiple references, including in [23, 24], have experimented with this compensation and concluded that it significantly reduces the impact of the current ripple. Therefore, it is imperative to ensure the implementation of dead-time compensation to optimize the performance of the inverter. A solution from obtained Pareto front is selected, comparing performances before and after installing the chosen motor set.

### Contributions

The complexity in this work is not only due to the mechanical dynamics of the two extra degrees of freedom, with a time-variant trajectory, but also the challenge of controlling BLDC motors.

This paper examines the dynamic behavior of torque, current, and voltage in a motor system during task operation. Unlike in traditional literature, this paper considers the mechanical system’s start current, non-linear effects, and acceleration changes between the motor, reducer, and load. The focus is on the dynamic profiles of these variables instead of assuming a continuous torque steady-state condition, the commonly used q-d transformation, or a priori known motor load torque. Therefore, three contributions of this article can be stated. First, a current reference generator is employed for the three-phase commutation conditions of motors. Thus, a three-dimensional current controller is used for each motor of the mechatronic system. It depends on the reference of current flow for the 60 degrees of commutation sequence instead of using a FOC control method (where sinusoidal voltages are obtained from the stationary system). Second, for the outer-loop controller, the trapezoidal function of BEMF is used to get a combined torque-position controller of the whole system. Third, a servo-amplifier is built to open the robot architecture to validate the proposed method on an experimental platform. We infer from the results that inertial contributions and control effects are distributed by evolutionary optimization while selecting motors, turning related constraints out to be feasible for the coupled mechanism, and reducing energy consumption.

The article is organized into six sections. **Dynamics of the Robotic System** presents the closed-loop dynamics of the overall system. **Operational Boundary Constraints** states operational constraints for the actuator selection process. **Multi-Objective Optimization Problem** establishes the multi-objective mixed-integer optimization problem. **Experimental Platform** presents experimental results for an industrial robot. Finally, **Conclusions** draw some concluding remarks.

## Dynamics of the robotic system

An industrial robot typically comprises the mechanism, the power train, and the controller. In this section, we present the dynamic modeling of each subsystem and the overall closed-loop dynamics.

### Mechanism dynamic model

Let us consider a rigid-link serial manipulator with *n* actuated joints, where $\text{q}\in R^{n}$ is the generalized coordinates vector. Hence, by using the Lagrangian formulation, [25], the robot dynamics can be written as

$$\begin{array}{c} \tau =\text{M}(\text{q})\ddot{\text{q}}+C\left(\text{q},\dot{\text{q}}\right)\dot{\text{q}}+\text{g}(\text{q}) \end{array}$$ (1)

where, $\text{M}(\text{q})\in R^{n\times n}$ is the inertia matrix, $\text{C}\left(\text{q},\dot{\text{q}}\right)\in R^{n\times n}$ is the Coriolis and centrifugal forces matrix, $\text{g}(\text{q})\in R^{n}$ is the gravity terms vector and $\tau \in R^{n}$ is the input torque vector. For simplicity, time dependence notation is omitted.

### Power-train dynamic model

**Fig 1:**
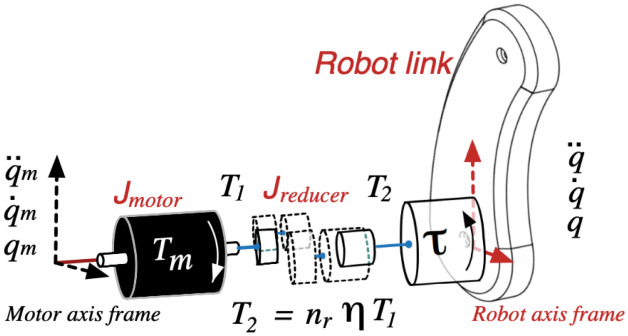
Power-train schematic at each joint.

A schematic for the powertrain at each joint is shown in Fig 1, where the BLDC motor exerts torque ***T***_***m***_(***t***), to drive a dynamic load ***τ***(***t***), with a given transmission ratio and efficiency, ***n***_***r***_ and ***η***, respectively. So, the powertrain dynamics for the ***k***-th joint are given by

$$\begin{array}{c} T_{m,k}\left(t\right)=J_{eq,k}n_{r,k}q_{k}+b_{eq,k}n_{r,k}{\dot{q}}_{k}+\frac{\tau_{k}\left(t\right)}{n_{r,k}\eta_{k}} \end{array}$$ (2)

where ***J***_***eq***,***k***_ and ***b***_***eq***,***k***_ are the equivalent inertia and viscous friction on the powertrain, obtained as

$$\begin{array}{c} J_{eq,k}=J_{motor,k}+J_{reducer,k} \end{array}$$ (3)

$$\begin{array}{c} b_{eq,k}=b_{motor,k}+b_{reducer,k} \end{array}$$ (4)

Considering all the robot joints, the whole power-train dynamics can be written as

$$\begin{array}{c} {\text{T}}_{\text{m}}\left(t\right)={\text{J}}_{eq}\Phi \ddot{\text{q}}+{\text{B}}_{eq}\Phi \dot{\text{q}}+{\left[\Phi ϒ\right]}^{-1}\tau \left(t\right)\; \end{array}$$ (5)

where **Φ** = diag(***n***_***r***,1_, …, ***n***_***r***,***n***_), **Υ** = diag(***η***_1_, …, ***η***_*n*_), ${\text{T}}_{\text{m}}\left(t\right)\in R^{n}$, $\tau \left(t\right)\in R^{n}$, **J**_***eq***_ = diag(***J***_***eq***,1_, …, ***J***_***eq***,***n***_), and **B**_***eq***_ = diag(***b***_***eq***,1_, …, ***b***_***eq***,***n***_).

Then, the combined robot and power-train dynamics can be expressed as

$$\begin{array}{c} {\text{T}}_{\text{m}}\left(t\right)=\text{H}(\text{q})\ddot{\text{q}}+D\left(\text{q},\dot{\text{q}}\right)\dot{\text{q}}+{\text{F}}_{R}\dot{\text{q}}+\text{G}(\text{q}) \end{array}$$ (6)

where

$$\begin{array}{c} \text{H}\left(\text{q}\right)=[{\text{J}}_{eq}\Phi +{\left(\Phi ϒ\right)}^{-1}\text{M}\left(\text{q}\right)]\; \end{array}$$ (7)

$$\begin{array}{c} \text{D}\left(\text{q},\dot{\text{q}}\right)=[{\left(\Phi ϒ\right)}^{-1}\text{C}\left(\text{q},\dot{\text{q}}\right)] \end{array}$$ (8)

$$\begin{array}{c} {\text{F}}_{\text{R}}=[{\text{B}}_{eq}\Phi ] \end{array}$$ (9)

$$\begin{array}{c} \text{G}\left(\text{q}\right)=[{\left(\Phi ϒ\right)}^{-1}\text{g}\left(\text{q}\right)]\; \end{array}$$ (10)

### Electrical dynamic model of actuators

The power-train is driven by BLDC motors, where the following assumptions are made, [13, 26].

**Fig 2:**
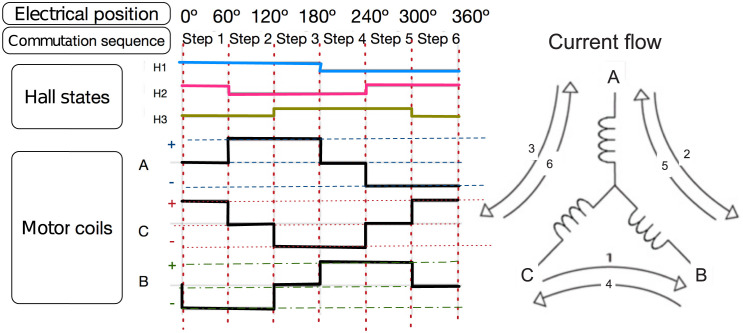
Commutation sequence of a BLDC motor.

- Each phase of the motor has an equal number of windings that are distributed symmetrically.
- According to [27], BLDC motors differ from other synchronous permanent magnet motors in that they have a Trapezoidal Back EMF instead of a sinusoidal one. Therefore, the Back Electromotive Force voltage (BEMF) is represented by a trapezoidal function dependent on the electrical winding position ***θ***_***e*,*k***_.
- There exists an electrical commutation sequence where the motor current must flow through its motor phases, as shown in Fig 2.
- The motor operates every 60 electrical degrees, and the Resistance and Inductance values for each phase can be simplified as described in the literature [28]. The three-phase armatures are assumed to be symmetrical to simplify these values, meaning ***R***_***a***_ = ***R***_***b***_ = ***R***_***c***_ = ***R***_***s***_, and ***L***_***a***_ = ***L***_***b***_ = ***L***_***c***_ = ***L***_***s***_, and the motor terminal voltage equation, for each joint, can be written as:
  $$\begin{array}{c} V_{a}=R_{s}I_{a}+L_{s}\frac{dI_{a}}{dt}+E_{a} \end{array}$$ (11)
  $$\begin{array}{c} V_{b}=R_{s}I_{b}+L_{s}\frac{dI_{b}}{dt}+E_{b} \end{array}$$ (12)
  $$\begin{array}{c} V_{c}=R_{s}I_{c}+L_{s}\frac{dI_{c}}{dt}+E_{c} \end{array}$$ (13)
  where ***V*** is the motor voltage, ***I*** is the motor current, ***E*** is the trapezoidal function ***E*** = ***f***(***θ***_***e***_) of the BEMF, and the subscript a,b, and c denotes the three phases of the motor, respectively.
- The eddy currents produced are directed only in the axial direction, meaning that end effects are ignored [29].
- A rigid connection between motor-reducer-load is assumed, and the following position relationship is fulfilled for the ***k***-th joint, ***θ***_***e***, ***k***_ = ***n***_***P***, ***k***_***n***_***r***, ***k***_***q***_***k***_, where ***θ***_***e***, ***k***_ is the electrical winding position and ***n***_***P***, ***k***_ the motor pair poles.

Considering the electrical dynamics of each motor as in 11–13, the electrical dynamics of all the installed motors on the manipulator can be re-written as

$$\begin{array}{c} {\dot{I}}_{z}={\text{L}}_{s}^{-1}\left[{\text{V}}_{z}-{\text{R}}_{s}I_{z}-{\text{E}}_{z}\right] \end{array}$$ (14)

$$\begin{array}{c} {\dot{\theta }}_{e}=\Theta \Phi \dot{\text{q}} \end{array}$$ (15)

$$\begin{array}{c} {\text{T}}_{\text{m}}=\Lambda \left[\sum_{z}T_{z}\right]\;,\;\text{for}\;z=\left\{1,2,3\right\}\; \end{array}$$ (16)

where for the ***z***-th phase, ${\text{I}}_{z}\in R^{n}$ is the motor current vector, ${\text{V}}_{z}\in R^{n}$ is the motor voltage input vector, ${\text{E}}_{z}\in R^{n}$ is the backEMF vector, and $T_{z}\in R^{n}$ is defined by its ***k***-th element as

$$\begin{array}{c} T_{z,k}=f_{z,k}\left(\theta_{e,k}\right)I_{z,k}\; \end{array}$$ (17)

Matrices **Θ** = diag(***n***_***P***,1_, …, ***n***_***P***,***n***_), **R**_**s**_ = diag(***R***_***s***,1_, …, ***R***_***s***,***n***_), **L**_**s**_ = diag{[***L***_***s***,1_, …, ***L***_***s***,***n***_]} and **Λ** = diag{[**λ**_1_, …, **λ**_***n***_]}, relate the total pole numbers, phase resistances, phase inductances, and induced flux amplitude, respectively. Function ***f***_***z***,***k***_(***θ***_***e***,***k***_), is the trapezoidal backEMF, [13], of the ***z***-th phase with ***θ***_***e***,***k***_ ∈ [**0**, **2*π***], related to the ***k***-th rotor position.

### Actuators control

**Fig 3:**
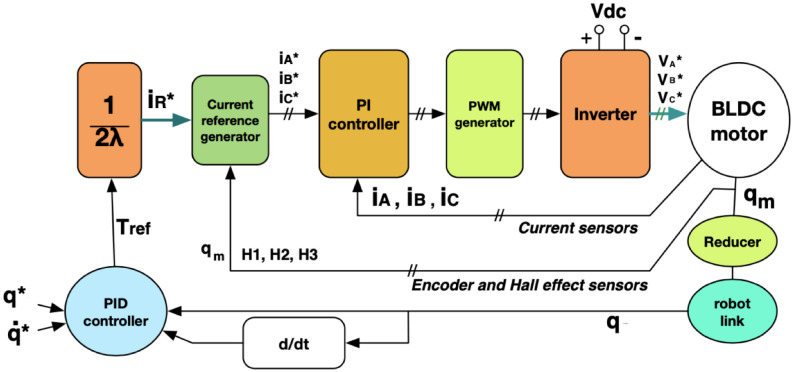
Torque-position control scheme for BLDC motors.

Fig 3 shows a control scheme for each BLDC motor, where each voltage phase input is the output of a PI controller. For all motors on the power train, Eqs (18) and (19) provide the voltage for phases A and B, respectively

$$\begin{array}{c} {\text{V}}_{\text{A}}^{*}=K_{p,A}\left({\text{I}}_{\text{A}}^{*}-{\text{I}}_{\text{A}}\right)+K_{I,A}\int_{0}^{T}\left({\text{I}}_{\text{A}}^{*}-{\text{I}}_{\text{A}}\right)dt \end{array}$$ (18)

$$\begin{array}{c} {\text{V}}_{\text{B}}^{*}={\text{K}}_{p,B}\left({\text{I}}_{\text{B}}^{*}-{\text{I}}_{\text{B}}\right)+{\text{K}}_{I,B}\int_{0}^{T}\left({\text{I}}_{\text{B}}^{*}-{\text{I}}_{\text{B}}\right)dt \end{array}$$ (19)

where **K**_***p***,***A***_, **K**_***p***,***B***_, **K**_***I***,***A***_ and **K**_***I***,***B***_ are diagonal definite positive matrices. Voltage ${\text{V}}_{\text{C}}^{*}$ can be obtained from motor’s electrical balance [13]. Desired currents ${\text{i}}_{R}^{*}$ depend on the current reference flow of the commutation sequence, whose values are given by

$$\begin{array}{c} {\text{i}}_{R}^{*}={\left[2*\Lambda \right]}^{-1}{\text{T}}_{\text{ref}} \end{array}$$ (20)

where **T**_**ref**_ is the desired motor torque vector and is the output of a PID controller intended to control the position tracking error.

$$\begin{array}{c} {\text{T}}_{\text{ref}}={\text{K}}_{1}\text{e}+{\text{K}}_{2}\dot{\text{e}}+{\text{K}}_{3}\int_{0}^{\text{T}}\text{e}\;dt \end{array}$$ (21)

where **K**_1_, **K**_2_, and **K**_3_ are diagonal definite positive matrices, **e** = (q* − **q**), and reference q* represents the desired vector of angular position.

### Mechatronic model of the robot

The closed-loop robot dynamics can be stated as

$$\begin{array}{c} \ddot{\text{q}}=\text{H}(\text{q}{)}^{-1}\left\{{\text{T}}_{\text{m}}-\text{D}\left(\text{q},\dot{\text{q}}\right)\dot{\text{q}}-{\text{F}}_{\text{R}}\dot{\text{q}}-\text{G}(\text{q})\right\} \end{array}$$ (22)

$$\begin{array}{c} {\dot{\text{I}}}_{\text{A}}={\text{L}}_{\text{s}}^{-1}\left[{\text{V}}_{\text{A}}^{*}-{\text{R}}_{\text{s}}{\text{I}}_{\text{A}}-{\text{E}}_{\text{A}}\right] \end{array}$$ (23)

$$\begin{array}{c} {\dot{\text{I}}}_{\text{B}}={\text{L}}_{\text{s}}^{-1}\left[{\text{V}}_{\text{B}}^{*}-{\text{R}}_{\text{s}}{\text{I}}_{\text{B}}-{\text{E}}_{\text{B}}\right] \end{array}$$ (24)

$$\begin{array}{c} {\dot{\text{I}}}_{\text{C}}={\text{L}}_{\text{s}}^{-1}\left[{\text{V}}_{\text{C}}^{*}-{\text{R}}_{\text{s}}{\text{I}}_{\text{C}}-{\text{E}}_{\text{C}}\right] \end{array}$$ (25)

$$\begin{array}{c} {\text{T}}_{\text{m}}=\Lambda \left[T_{\text{A}}+T_{\text{B}}+T_{\text{C}}\right] \end{array}$$ (26)

$$\begin{array}{c} {\dot{\theta }}_{e}=\Theta \Phi \dot{\text{q}} \end{array}$$ (27)

where for each ***k***-th motor of the robot

$$\begin{array}{c} T_{A,k}=f_{A,k}\left(\theta_{A_{e},k}\right)I_{A,k}\; \end{array}$$ (28)

$$\begin{array}{c} T_{B,k}=f_{B,k}\left(\theta_{B_{e},k}\right)I_{B,k}\; \end{array}$$ (29)

$$\begin{array}{c} T_{C,k}=f_{C,k}\left(\theta_{C_{e},k}\right)I_{C,k}\; \end{array}$$ (30)

## Operational boundary constraints

The proposed methodology considers *off-the-shelf* motor selection, assuming that motor parameters (such as masses, inertias, resistances, inductances, etc.) and boundary values for safe motor operation are available in manufacturers’ catalogs. Such boundary values are used as feasibility constraints to verify that each motor’s dynamic response stays within its safe operation zone as suggested in [30].

- The Continuous operation zone limit, constrained by the steady-state motor torque, where ***t***_***f***_ is the time cycle of the required task,
  $$\begin{array}{c} T_{M,N}\geq T_{M,rms}=\sqrt{\frac{1}{t_{f}}\int_{0}^{t_{f}}{\left\{T_{m}\left(t\right)\right\}}^{2}dt} \end{array}$$ (31)
- The Dynamic operation zone limit, constrained by the maximum torque,
  $$\begin{array}{c} T_{M,max}\geq \|T_{m}{\|}_{\infty }=\text{max}\{|T_{m}\left(t\right)\left|\right\} \end{array}$$ (32)
- The Maximum range of velocity, constrained by the maximum motor velocity,
  $$\begin{array}{c} \omega_{M,max}\geq \|\omega_{m}{\|}_{\infty }=\text{max}\{|\dot{q}\left(t\right)\cdot n_{r}\left|\right\} \end{array}$$ (33)
- Rated current limit, constrained by the steady-state driving current,
  $$\begin{array}{c} I_{N,M}\geq \sqrt{\frac{1}{t_{f}}\int_{0}^{t_{f}}\{\frac{T_{m}\left(t\right)}{2\lambda }{\}}^{2}dt} \end{array}$$ (34)
- Peak current limit, constrained by the maximum driving current,
  $$\begin{array}{c} I_{max}\geq \text{max}\{|\frac{T_{m}\left(t\right)}{2\lambda }|\} \end{array}$$ (35)
- Peak induced limit voltage by inverter ***V***_***BUS***_, constrained by the maximum dynamic range of motor voltage
  $$\begin{array}{c} V_{max}\geq \text{max}\left\{|V_{BUS}|\right\} \end{array}$$ (36)
- Transmission requirements may be considered, such as maximum load on the transmission ***T***_***G***,***max***_, and the maximum input velocity to the reducer ***ω***_***G***,***max***_,
  $$\begin{array}{c} T_{G_{i},max}\geq {\left\|\tau \right\|}_{\infty }=\text{max}\left\{|\tau \left(t\right)|\right\} \end{array}$$ (37)
  $$\begin{array}{c} \omega_{G,max}\geq \|\omega_{m}{\|}_{\infty }=\text{max}\{|\dot{q}\left(t\right)\cdot n_{r}\left|\right\} \end{array}$$ (38)

We also consider changes in inertial parameters when testing motors on the mechanism. Thus, the following changes are regarded for each ***i***-th local frame.

$$\begin{array}{c} \text{Total}\;\text{mass}\;\text{contribution:}\;m_{i}=\sum_{j=1}^{p}m_{i,j} \end{array}$$ (39)

$$\begin{array}{c} \text{Mass}\;\text{center}\;\text{location:}\;{\text{O}}_{\text{ci}}=\frac{1}{m_{i}}\sum_{j=1}^{p}{\text{O}}_{c_{\text{i},\text{j}}}m_{i,j} \end{array}$$ (40)

$$\begin{array}{c} \text{Total}\;\text{Inertia:}\;{\text{I}}_{\text{i}}=\sum_{j=1}^{p}\left\{{\text{I}}_{\text{i},\text{j}}+m_{i,j}{\text{S}}^{\text{T}}\left({\text{r}}_{\text{i},\text{j}}\right)\text{S}\left({\text{r}}_{\text{i},\text{j}}\right)\right\} \end{array}$$ (41)

where ***m***_***i***,***j***_, ${\text{O}}_{{\text{c}}_{\text{i},\text{j}}}$ and **I**_**i,j**_, are the mass contribution, mas center location, and invariant inertia tensor for the ***j***-th element involved at the local frame respectively. Integer ***p*** represents the total quantity of parts involved at each *i*-th local frame, and **S**(**r**_**i,j**_) is the skew symmetric matrix associated with the position vector ${\text{r}}_{\text{i},\text{j}}=\left({\text{O}}_{{\text{c}}_{\text{i},\text{j}}}-{\text{O}}_{\text{ci}}\right)$.

## Multi-objective optimization problem

This paper focuses on minimizing three objective functions for the robot’s motor selection, energy consumption, tracking error, and motors’ total weight. There is a conflict among the three objective functions. If lower energy consumption is desired, the error in trajectory tracking can be increased, or the motors’ weight can be reduced since their weight is proportional to energy consumption. However, if the error in trajectory tracking is reduced, the controller gains must be increased, which leads to higher energy consumption. Alternatively, larger motors with greater torque capacities can be selected, but it must be ensured that no system constraints are violated, such as starting currents or current peaks. This section details the objective functions as well as the design decision variables.

### Objective functions

#### Energy consumption

Let us consider that ***P***_***M***,***k***_ is the power consumption of the ***k***-th motor on the mechanism, given by the sum of electrical ***W***_***E***,***k***_, and mechanical ***W***_***M***,***k***_ power provided by such motor, as

$$\begin{array}{c} W_{E,k}=R_{s,k}I_{m}^{2}\;,\;k=1,...,n \end{array}$$ (42)

$$\begin{array}{c} W_{M,k}=T_{m,k}n_{r,k}{\dot{q}}_{k}\;,\;k=1,...,n \end{array}$$ (43)

Then, the total power consumption to drive the robot is

$$\begin{array}{c} P_{\text{M}}={\text{W}}_{\text{E}}+{\text{W}}_{\text{M}} \end{array}$$ (44)

where ${\text{P}}_{\text{M}}\in R^{n}$, ${\text{W}}_{\text{E}}\in R^{n}$, and ${\text{W}}_{\text{M}}\in R^{n}$. The objective function to minimize the energy consumption is stated as

$$\begin{array}{c} f_{1}=\int_{0}^{t_{f}}{\text{P}}_{\text{M}}{\left(t\right)}^{T}{\text{P}}_{\text{M}}\left(t\right)dt \end{array}$$ (45)

under the optimal set of motors and control gains.

#### Tracking error

The tracking error objective function is given by

$$\begin{array}{c} f_{2}=\int_{0}^{t_{f}}{\text{e}}^{T}\text{e}dt\;. \end{array}$$ (46)

#### Total weight of motors

The objective is to minimize the total weight of actuators by selecting the lowest weight motors able to perform the required task, i.e.

$$\begin{array}{c} f_{3}=\sum_{i=1}^{n}m_{m,i} \end{array}$$ (47)

where ***m***_***m***,***i***_ is the *i*-th motor mass of the robot.

### Decision variables

#### Discrete decision vector for the selection process

For the ***k***-th actuated joint, an integer index ***z***_***m***,***k***_ is used to select a motor from a list of ***d***_***m***_ candidate motors. Then, if the robot has ***n*** active joints to be driven by selected motors, the integer decision vector is

$$\begin{array}{c} {\text{z}}_{\text{m}}\in Z^{n}\;,\;\text{where}\;1\leq z_{m,k}\leq d_{m} \end{array}$$ (48)

#### Continuous decision vector for the control process

Considering the proposed control scheme in Fig 3, the ***k***-th actuated joint is required to tune seven control gains, corresponding to the ***k***-th diagonal positive elements of the matrices **K**_**1**_, **K**_**2**_, **K**_**3**_, **K**_***p***,***A***_, **K**_***p***,***B***_, **K**_***I***,***A***_, and **K**_*I*,*B*_, respectively. Thus, if the robot has *n* active joints, the whole controller gains make up the continuous decision vector **x**_**c**_, stated as

$$\begin{array}{c} x_{\text{c}}\in R^{7\times n}\;,\;\text{where}\;x_{c,i}>0 \end{array}$$ (49)

### Concurrent optimization problem

A multi-objective dynamic optimization problem is finding the decision variable vector $X^{*}=[{\text{z}}_{\text{m}}^{*},x_{\text{c}}^{*}{]}^{\text{T}}$ that optimizes the objective function vector to select and control the robot actuators optimally [31].

$$\begin{array}{c} \underset{\text{X}}{\text{min}}\;\text{F}=\left[f_{1},f_{2},f_{3}\right] \end{array}$$ (50)

subject to:

- The closed-loop dynamics of the robot (22)–(27).
- The **8** × ***n*** motors’ feasibility constraints (31)–(38).
- The **3** × ***n*** inertial parameters’ changes (39)–(41).

### Genetic algorithm NSGA-II

**Fig 4:**
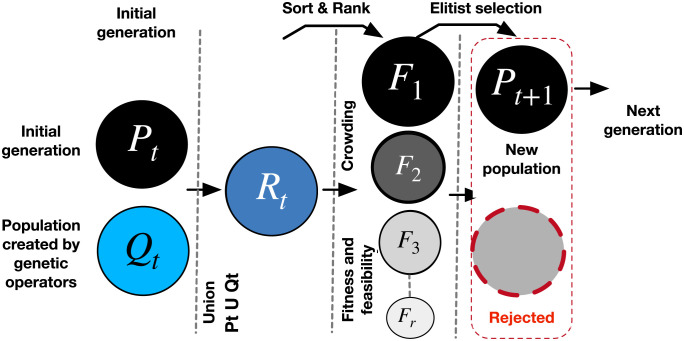
One iteration of the NSGA-II algorithm.

The genetic algorithm NSGA-II, [32], is used to obtain the Pareto-Front of the proposed multi-objective optimization problem since it could find diversity and spread of non-dominated solutions compared to other meta-heuristics. For this reason, NSGA-II has been selected in this study as an optimization solver [33, 34]. A flowchart of an iteration is shown in Fig 4 as described in the following steps 1–10.

1. First generation, an initial population ***P***_***t***_(***N***) is randomly created, with ***N*** chromosomes, where each chromosome contains a set of decision variables $X^{\text{i}}=[{\text{z}}_{\text{m}}^{\text{i}},x_{\text{c}}^{\text{i}}{]}^{\text{T}}$.
2. Verify the feasibility of constraints (22–41) and evaluate each objective function (45, 46, 47) obtaining the *fitness* vector $\left[f_{1}^{i},f_{2}^{i},f_{3}^{i}\right]$ from ***P***_***t***_(***N***).
3. Sort ***P***_***t***_(***N***) according to dominance to generate ***r*** fronts and rank. Also, apply crowding distance calculation to verify spread of population.
4. A population ***M***_***t***_(***N***) is obtained from the selection by tournament(Crowded Binary Tournament Selection) of the population ***P***_***t***_(***N***).
5. A new population ***Q***_***t***_(***M***) is created from ***M***_***t***_(***N***) by applying genetic algorithm operators. The crossover (two-point crossover) is the primary operator prioritized by the Genetic Algorithm, while the mutation (bit inversion) is the secondary operator.
6. Verify the feasibility of constraints (22–41) and evaluate each objective function (45, 46, 47) obtaining the *fitness* vector $\left[f_{1}^{i},f_{2}^{i},f_{3}^{i}\right]$ from ***Q***_***t***_(***M***).
7. Merge ***P***_***t***_(***N***) and ***Q***_***t***_(***M***) to create ***R***_***t***_ = ***P***_***t***_ ⋃ ***Q***_***t***_.
8. Sort ***R***_***t***_(***N*** + ***M***) according to dominance to generate ***r*** fronts and rank as ***F***_**1**_, ***F***_**2**_, ***F***_**3**_, …, ***F***_***r***_. Also, apply crowding distance calculation to verify spread of population.
9. Select the best population from ***R***_***t***_(***N*** + ***M***) and assigned it to ***P***_***t*+1**_ until ***size***(***P***_***t*+1**_) = ***N***.
10. Go to next generation (step 4), until the maximum number of generations is attained.

## Experimental platform

As a case study, to illustrate the performance of our proposal, we consider a Samsung industrial robot model Faraman AT1 driven by three-phase BLDC servo motors. The parameters for nominal (originally installed) robot motors are shown within the list of candidate motors in Tables 5 and 6 in S1 Appendix, where the initial vector of indexes for nominal motors is **z**_***m***_ = [**22**, **22**, **21**, **36**, **36**].

**Fig 5:**
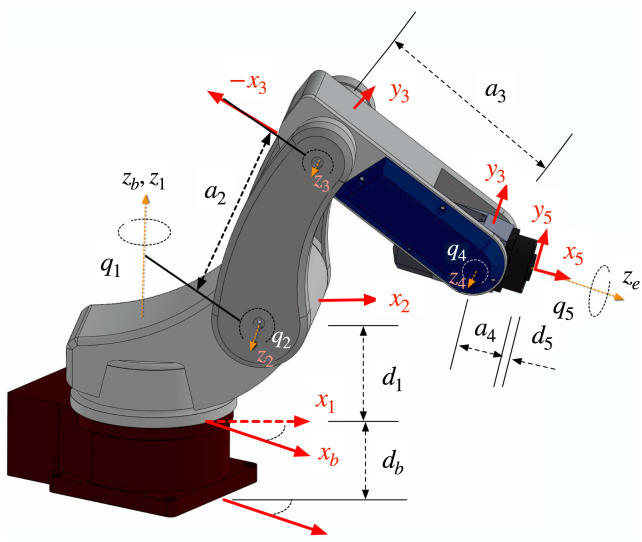
Robot configuration.

Fig 5, shows the robot kinematic configuration, where ***d***_**1**_ = **0.125** [***m***], ***d***_**2**_ = **0.17** [***m***], ***a***_**1**_ = **0.17** [***m***], ***a***_**2**_ = **0.26** [***m***], ***a***_**3**_ = **0.27** [***m***], ***a***_**4**_ = **0.069** [***m***] and ***d***_**5**_ = **0.01** [***m***]. Inertial parameters were obtained via CAD tools. The nominal reducers’ transmission ratios are ***n***_***r***,**1**_ = **120**, ***n***_***r***,**2**_ = **120**, ***n***_***r***,**3**_ = **100**, ***n***_***r***,**4**_ = **80** and ***n***_***r***,**5**_ = 50.

The desired task has the following conditions

- Helical shape with center location, ***c***(***x***, ***y***, ***z***) = [**0.519**, **0**, **0.555**] [***m***], radius ***r*** = **0.15** [***m***], and depth ***P***_***rf***_ = **0.1** [***m***].
- Orientation during the task, ***roll*** = **0** and ***pitch*** = **0** [***rad***].
- Initial position, ***P***_1_(***x***, ***y***, ***z***) = [**0.519, 0.15, 0.555**]^***T***^ [***m***].
- Cycle time ***t***_***f***_ = **35**[***s***].
- Required tracking error, less than **0.1** [***rad***].

**Fig 6:**
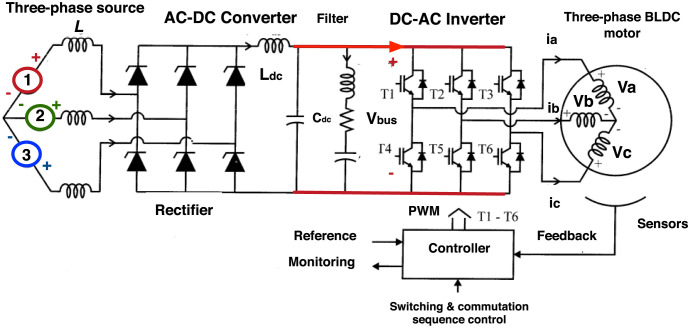
Inverter scheme of each joint.

In order to apply the proposed method with actual data, a servo-amplifier was built to open the robot architecture. Each motor inverter in the servo-amplifier was built based on the scheme shown in Fig 6.

The robot can drive a maximum load of 3 [Kg] (contracted arm). A tool was installed at the end effector to carry out the desired task, and the nominal motors should operate over their limits.

**Fig 7:**
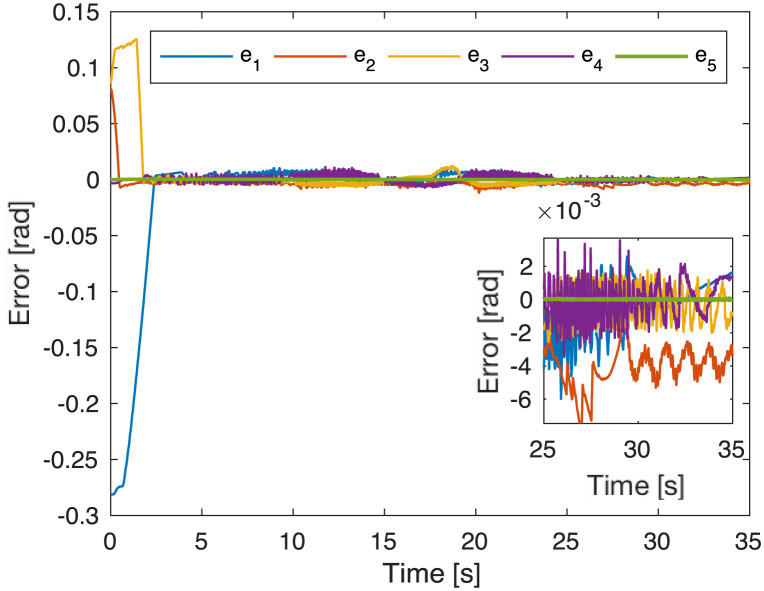
Tracking error of the desired task.

Experimental tests were performed until the tracking error was bounded between +/- 0.02 [rad] and motors reached the desired references, as shown in Fig 7. However, as expected to maintain such performance with desired velocity and load, joints 1 and 3 exceed the limits for safe operation.

For comparison reasons, the obtained response is named a “nominal solution”. Thus, the control gain values for the nominal solution are presented in Table 1.

**Table 1:** Control gain values of nominal solution.

Joint	*K*_*P*,*A*_	*K*_*I*,*A*_	*K*_*P*,*B*_	*K*_*I*,*B*_	*K*_1_	*K*_2_	*K*_3_
1	12	0.3	12	0.3	18	0.01	0.0001
2	28	0.3	28	0.3	16	0.01	0
3	34	2	34	2	19	0.2	0.03
4	20	3	20	3	22	1.5	0
5	35	3	35	3	60	1.5	0

It is noticed that controller gain tuning and actuator selection process optimize concurrent objectives. The motor’s weight is proportional to the energy consumption required to drive a load (the lighter motor, the lower the torque-velocity profile required). Moreover, installing another motor set changes the robot’s inertia and mass distribution, then gain tuning is to be set again to improve the tracking error.

### Statement of the optimization problem

A list of 37 BLDC-motors is used, where parameters and limits are shown in Tables 5 and 6 in S1 Appendix. Thus, concurrent optimization problem can be stated as follows.

It is required to find a decision vector of the form ${\text{x}}^{*}=[{\text{z}}_{m}^{*},{\text{x}}_{c}^{*}{]}^{T}$ in order to optimize the objective function vector

$${\text{min}}_{\text{x}}\;F\left(\text{X}\right)=[\sum_{k=1}^{5}m_{m,k},\int_{0}^{t_{f}}\text{e}{\left(t\right)}^{T}\text{e}\left(t\right)dt,\int_{0}^{t_{f}}{\text{P}}_{\text{M}}{\left(t\right)}^{T}{\text{P}}_{\text{M}}\left(t\right)dt{]}^{T}$$

where ${\text{z}}_{m}^{*}\in Z^{5}$ and ${\text{x}}_{c}^{*}=\in R^{35}$, subject to **30** constraints,

$$\begin{array}{c} T_{M_{k},max}\left(\text{x}\right)\geq \text{max}\{|T_{m,k}\left(\text{x},t\right)\left|\right\}\\ T_{M_{k},N}\left(\text{x}\right)\geq \sqrt{\frac{1}{t_{f}}\int_{0}^{t_{f}}{\left\{T_{m,k}\left(\text{x},t\right)\right\}}^{2}dt}\\ \omega_{M_{k},max}\left(\text{x}\right)\geq \text{max}\{|n_{r,k}\left(\text{x}\right)\cdot {\dot{q}}_{k}\left(\text{x},t\right)\left|\right\}\\ I_{N,M_{k}}\left(\text{x}\right)\geq \sqrt{\frac{1}{t_{f}}\int_{0}^{t_{f}}\{\frac{T_{m,k}\left(\text{x},t\right)}{2\lambda_{p,k}\left(\text{x}\right)}{\}}^{2}dt}\\ I_{max,k}\left(\text{x}\right)\geq \text{max}\{|\frac{T_{m,k}\left(\text{x},t\right)}{2\lambda_{p,k}\left(\text{x}\right)}|\}\\ T_{G_{k},max}\left(\text{x}\right)\geq \text{max}\{|\tau_{k}\left(\text{x},t\right)\left|\right\}\\ 1\leq z_{m,k}\leq 37\;,\;x_{c,l}\geq 0\;\\ k=1,\ldots ,5\;,\;l=1,\ldots ,35 \end{array}$$

subject to the **3** × **5** inertial parameters’ changes (39)–(41), and the dynamic response of the closed-loop system given by (22)–(27).

## Results

**Fig 8:**
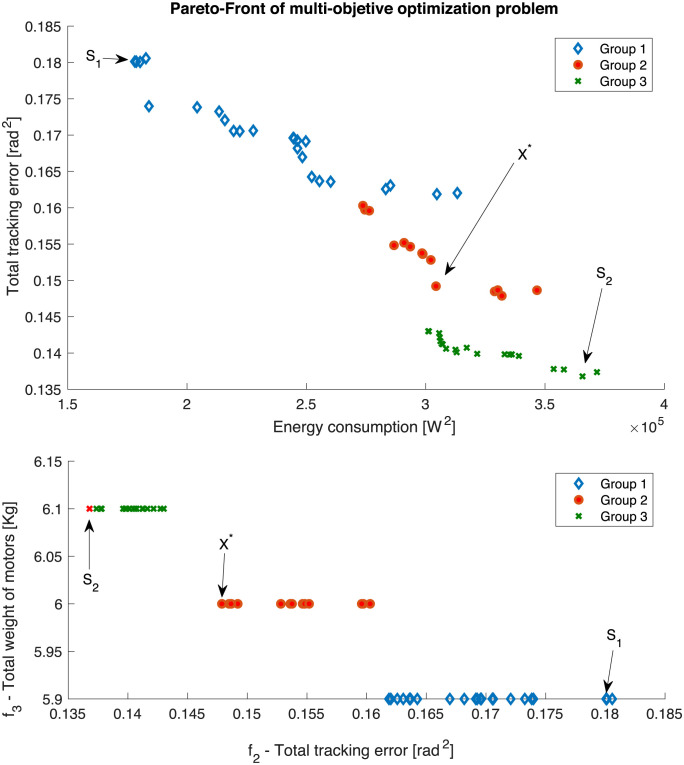
Pareto-Front of the multi-objective optimization problem. The non-dominated solutions are classified into Group 1, Group 2, and Group 3.

The NSGA-II Algorithm is used to solve the previously stated optimization problem with a population size of 100 chromosomes. Fig 8 shows the resulting Pareto front, where it is observed that three different groups of motors are obtained, where their total motors’ weight matched between them. Group 1 is represented by a blue diamond, Group 2 by a red dot, and Group 3 by a green cross. It is observed from Fig 8 that the lower the energy consumption (***f***_**1**_), the higher the tracking error(***f***_**2**_). In addition, the weight of motors is proportional to their energetic capacity to drive a given load. For this reason, the heavier the motor (***f***_**3**_), the lower the tracking error (***f***_2_). Moreover, for a sum of weights of the selected motors (***f***_**3**_), for example, all the solutions of group 1, the variation in the second objective function is due to the different controller gains proposed by NSGA-II Algorithm.

- Group 1: **z**_***m***_ = [**22, 22, 20, 36, 35**]
- Group 2: **z**_***m***_ = [**22, 22, 20, 36, 36**]
- Group 3: **z**_***m***_ = [**22, 22, 20, 37, 36**]

Solutions named ***S***_1_ and ***S***_2_ indicate both extremes of the Pareto front and are presented in Table 2. Considering trade-offs among objective performances, a non-dominated solution X* is subjectively selected as it seems to be equally weighted among objectives.

**Table 2:** Comparative fitness.

Solution	*f*_1_ [*W*^2^]	*f*_2_ [*rad*^2^]	*f*_3_ [*Kg*]
***S*_1_**	**1.78 × 10^5^**	**0.180**	**5.9**
***S*_2_**	**3.65 × 10^5^**	**0.136**	**6.1**
X*	**3.04 × 10^5^**	**0.149**	**6**

The selected chromosome of the non-dominated solution contains the following values:

-
${\text{z}}_{m}^{*}=[22,22,20,36,36]$ .
- Table 3 shows the control gains’ values.

**Table 3:** Control gain values for the selected solution.

Joint	*K*_*P*,*A*_	*K*_*I*,*A*_	*K*_*P*,*B*_	*K*_*I*,*B*_	*K*_1_	*K*_2_	*K*_3_
1	52.56	15.71	52.56	15.71	58.96	0.05	0.31
2	68.66	1.42	68.66	1.42	79.45	0.05	0.62
3	49.71	0.34	49.71	0.34	59.65	0.97	0.45
4	98.85	9.08	98.85	9.08	65.98	0.41	18.09
5	66.67	37.58	66.67	37.58	81.57	0.08	4.072

Table 4 shows a quantitative comparison between nominal and selected solutions.

**Table 4:** Comparison between nominal and selected solutions.

**Performance of nominal solution**					
Joint	***f***_**1**_ [***W***^**2**^]	***f***_**2**_ [***rad***^**2**^]	***f***_**3**_ [***Kg***]	***P***_***rms***_ [***W***]	Constraints satisfied?
1	2574	0.1030	1.8	8.5425	No
2	7261	0.0021	1.8	14.386	Yes
3	2441	0.0344	0.8	8.342	No
4	178.766	**2.91** × **10**^−4^	0.4	2.268	Yes
5	0.0181	**1.02** × **10**^−6^	0.4	0.0227	Yes
Total	**12455.4**	**0.13979**	**5.2**	**33.562**	
**Performance of selected non-dominated solution**					
Joint	***f***_**1**_ [***W***^**2**^]	***f***_**2**_ [***rad***^**2**^]	***f***_**3**_ [***Kg***]	***P***_***rms***_ [***W***]	Constraints satisfied?
1	3266	0.0484	1.8	9.574	Yes
2	5709	0.0017	1.8	12.783	Yes
**3**	**1286.8**	**0.0092**	**1.6**	**6.0550**	**Yes**
4	77.691	**2.27** × **10**^−4^	0.4	1.49	Yes
5	0.016	**3.17** × **10**^−7^	0.4	0.0092	Yes
**Total**	**10340.6**	**0.0595**	**6**	**29.919**	
**Improvement**	↓ **16.97%**	↓ **57.41%**	↑ **15.38%**	↓ **10.85%**	

Note that the selected solution’s control gains (Table 3) are higher than nominal (Table 4). However, such a solution is feasible, as seen in the last column of Table 4. It can be explained by the fact that chosen motor has around four times wider torque-velocity operating range to drive the third-joint load, weighing just two times the nominal motor’s weight, having this joint the highest requested torque. Therefore, not only is a light motor selection essential but also its torque and energy capabilities.

**Fig 9:**
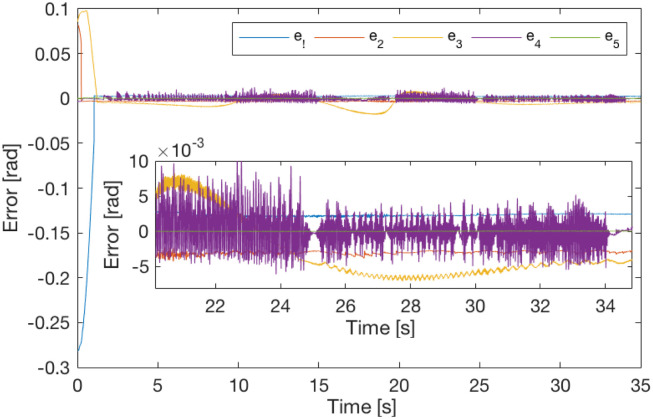
Tracking error for the selected solution.

**Fig 10:**
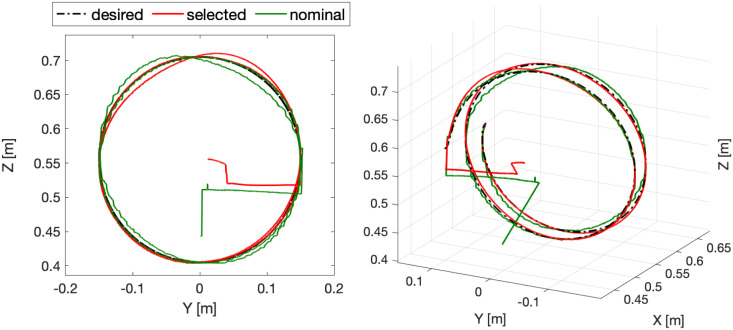
Obtained trajectory in Cartesian space.

**Fig 11:**
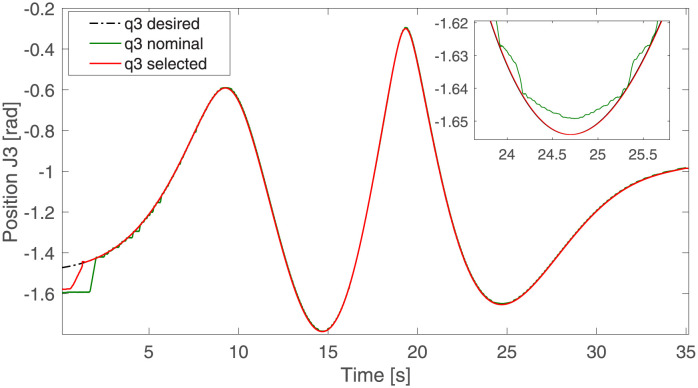
Third joint, angular position.

Table 4 demonstrates that the squared power consumption and the consumed electrical power of the non-dominated solution are reduced by 16.97% and 10.85%, respectively, but the robot’s weight increased by 15.3% compared to the nominal solution. The most significant advantage is generated in the trajectory tracking error since it is reduced by 57.41% using the proposed methodology versus the nominal solution; this effect can be seen in Fig 9, where the tracking errors are bounded around +/− **0.02** [***rad***^**2**^] once the motor achieves desired references. Another consequence of the increase in the proposed controller gains in the non-dominated and nominal solutions can be observed when comparing the position error for each degree of freedom. The settling times of the non-dominated solution (Fig 9) are usually shorter than those of the nominal solution (Fig 7). This shorter settling time between the non-dominated and nominal solution can be seen at the end effector trajectory differences in the Cartesian space in Fig 10 and the transient time in Fig 11.

Since the third motor changes with the non-dominated solution versus the nominal one, it has been selected as the degree of freedom to exemplify the dynamics of the whole system. Fig 11 shows the desired trajectory as a solid black line. The nominal solution is shown in red, while the selected non-dominated solution is in red. The zoomed box of this figure shows how the angular position of the nominal solution fluctuates around the desired trajectory. Likewise, it is observed that the non-dominated solution presents a smoother behavior than the nominal solution with less error to the desired trajectory.

**Fig 12:**
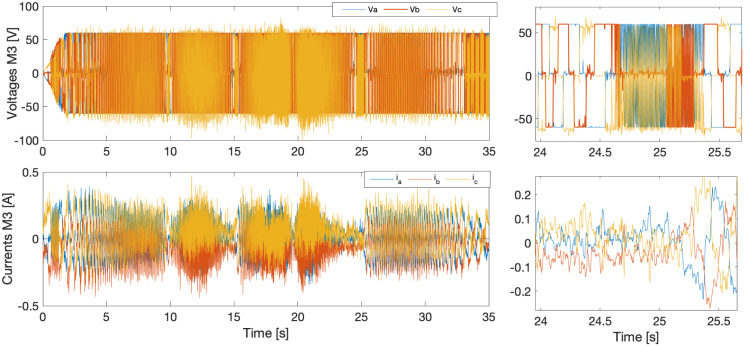
Third joint, voltage and current.

Fig 12 depicts the voltage and current of phase an in blue, phase b in red, and phase c in yellow. In the zoomed areas, it can be seen the non-sinusoidal transition of the currents and voltages of the selected motor. In addition, it can be seen in the current graph that in the time intervals of 10–15, 15–20, and 20–25, there is a frequency increase due to changes in the directions of the motor, as can be contrasted with Fig 11.

## Conclusions

A concurrent optimization method is proposed for the selection and control of BLDC motors, which are tested on a real industrial robot, obtaining feasible and non-dominated solutions for the required task.

A three-phase electrical dynamics of AC servomotors are proposed for BLDC motors in the mechatronic model of the powertrain for industrial robots. In this case, the RMS power of BLDC motors is computed to compare the energy consumption between the nominal and selected solution. While in the proposed method for PMSM motors, the nominal current on the powertrain is defined by Clark’s and Park’s transformation, ***q*** − ***d***, stationary systems that tend to a constant value to yield convergence of the voltages of the transformed quadrature. In the case study, a reduction of energy consumption of around **16.97%** is obtained. Even when the total weight of motors is higher for the selected non-dominated solution, it was possible, due to simulations of the closed-loop system, to analyze actuators’ limits and ranges to fulfill constraints. Moreover, it is essential to consider inertial changes due to different actuators since the system response also depends on such changes.

Furthermore, this proposal is applied to the actuators’ selection for a robot to follow a specific desired trajectory, which means that actuators’ selection and control gains values are simultaneously settled for that required task. Then, the proposed methodology allows for obtaining feasible, real, and viable solutions to perform a desired task satisfying concurrent objectives for an industrial robot.

## Supporting information

S1 Appendix (PDF)

## References

[1] Park JH, Asada H. Concurrent design optimization of mechanical structure and control for high speed robots. Journal of Dynamics Systems, Measurement and Control. 1994;4(3):344–356. doi: 10.1115/1.2899229

[2] Rastegar JS, Liu L, Yin D. Task-specific optimal simultaneous kinematic, dynamic, and control design of high-performance robotic systems. IEEE/ASME transactions on mechatronics. 1999;4(4):387–395. doi: 10.1109/3516.809517

[3] Villarreal-Cervantes MG, Cruz-Villar CA, Alvarez-Gallegos J, Portilla-Flores EA. Robust structure-control design approach for mechatronic systems. IEEE/ASME Transactions on Mechatronics. 2012;18(5):1592–1601. doi: 10.1109/TMECH.2012.2208196

[4] Cao Y, Liu Y, Ye X, Zhao J. An Automated Approach for Execution Sequence-Driven Software and Physical Co-Design of Mechatronic Systems Based on Hybrid Functional Ontology. Computer-Aided Design. 2021;131:102942. doi: 10.1016/j.cad.2020.102942

[5] Pettersson M, Ölvander J. Drive train optimization for industrial robots. IEEE Transactions on Robotics. 2009;25(6):1419–1424. doi: 10.1109/TRO.2009.2028764

[6] Zhou L, Bai S. A new approach to design of a lightweight anthropomorphic arm for service applications. Journal of Mechanisms and Robotics. 2015;7(3):031001. doi: 10.1115/1.4052699

[7] Singla E, Singh S, Singla A. Drive-train selection criteria for n-dof manipulators: basis for modular serial robots library. International Journal of Nonlinear Sciences and Numerical Simulation. 2021;22(2):169–181. doi: 10.1515/ijnsns-2017-0270

[8] Reyer JA, Papalambros PY. Combined optimal design and control with application to an electric DC motor. J Mech Des. 2002;124(2):183–191. doi: 10.1115/1.1460904

[9] Padilla-Garcia EA, Rodriguez-Angeles A, Resendiz JR, Cruz-Villar CA. Concurrent optimization for selection and control of AC servomotors on the powertrain of industrial robots. IEEE Access. 2018;6:27923–27938. doi: 10.1109/ACCESS.2018.2840537

[10] Zhang W, Xu Y, Zhou G. Research on a novel transverse flux permanent magnet motor with hybrid stator core and disk-type rotor for industrial robot applications. IEEE Transactions on Industrial Electronics. 2020;68(11):11223–11233. doi: 10.1109/TIE.2020.3038060

[11] Pillay P, Krishnan R. Application characteristics of permanent magnet synchronous and brushless DC motors for servo drives. IEEE Transactions on industry applications. 1991;27(5):986–996. doi: 10.1109/28.90357

[12] Nikam A, Jadhav H. Modelling & Simulation of Three Phases BLDC Motor for Electric Braking. In: 2019 2nd International Conference on Intelligent Computing, Instrumentation and Control Technologies (ICICICT). vol. 1. IEEE; 2019. p. 540–544.

[13] Krishnan R. Permanent magnet synchronous and brushless DC motor drives. CRC press; 2017.

[14] Hasanhendoei GR, Afjei E, Naseri M, Azad S. Automatic and Real Time Phase Advancing in BLDC Motor by Employing an Electronic Governor for a Desired Speed-Torque/Angle Profile. e-Prime-Advances in Electrical Engineering, Electronics and Energy. 2023; p. 100111.

[15] Bharanigha V, Shuaib YM. Minimization of torque ripples with optimized controller based four quadrant operation & control of BLDC motor. Advances in Engineering Software. 2022;172:103192. doi: 10.1016/j.advengsoft.2022.103192

[16] Rajesh A, Saravanan AG. Torque ripple minimization of bridgeless CUK converter-based BLDC motor using Improved Jellyfish Search Algorithm. ISA transactions. 2023;136:374–389. doi: 10.1016/j.isatra.2022.11.025 36535836

[17] Prathibanandhi K, Yaashuwanth C, Basha AR. Improved torque performance in BLDC-motor-drive through Jaya optimization implemented on Xilinx platform. Microprocessors and Microsystems. 2021;81:103681. doi: 10.1016/j.micpro.2020.103681

[18] Bharathi M, et al. Extraction of maximum power from solar with BLDC motor driven electric vehicles based HHO algorithm. Advances in Engineering Software. 2022;170:103137. doi: 10.1016/j.advengsoft.2022.103137

[19] Soori M, Arezoo B, Dastres R. Optimization of Energy Consumption in Industrial Robots, A Review. Cognitive Robotics. 2023;. doi: 10.1016/j.cogr.2023.05.003

[20] Meoni F, Carricato M. Optimal selection of the motor-reducer unit in servo-controlled machinery: A continuous approach. Mechatronics. 2018;56:132–145. doi: 10.1016/j.mechatronics.2018.11.002

[21] Boscariol P, Richiedei D. Energy optimal design of servo-actuated systems: A concurrent approach based on scaling rules. Renewable and Sustainable Energy Reviews. 2022;156:111923. doi: 10.1016/j.rser.2021.111923

[22] Wang Y, Niu S, Fu W. Sensitivity analysis and optimal design of a dual mechanical port bidirectional flux-modulated machine. IEEE Transactions on Industrial Electronics. 2017;65(1):211–220. doi: 10.1109/TIE.2017.2719620

[23] Shen Z, Jiang D. Dead-Time Effect Compensation Method Based on Current Ripple Prediction for Voltage-Source Inverters. IEEE Transactions on Power Electronics. 2019;34(1):971–983. doi: 10.1109/TPEL.2018.2820727

[24] Mannen T, Fujita H. Dead-Time Compensation Method Based on Current Ripple Estimation. IEEE Transactions on Power Electronics. 2015;30(7):4016–4024. doi: 10.1109/TPEL.2014.2352716

[25] Siciliano B, Sciavicco L, Villani L, Oriolo G. Kinematics. Robotics: Modelling, Planning and Control. 2009; p. 39–103.

[26] Baldursson S. Bldc motor modelling and control-a matlab^®^/simulink^®^ implementation. Chalmers University of Technology; 2005.

[27] Sakunthala S, Kiranmayi R, Mandadi PN. A study on industrial motor drives: Comparison and applications of PMSM and BLDC motor drives. In: 2017 International Conference on Energy, Communication, Data Analytics and Soft Computing (ICECDS); 2017. p. 537–540.

[28] Rambabu S. Modeling and control of a brushless DC motor. Master of Thesis In Power Control and Drives Technology, National Institute of Technology Rourkela. 2007.

[29] Ishak D, Zhu Z, Howe D. Eddy-current loss in the rotor magnets of permanent-magnet brushless machines having a fractional number of slots per pole. IEEE Transactions on magnetics. 2005;41(9):2462–2469. doi: 10.1109/TMAG.2005.854337

[30] Cusimano G, Casolo F. An almost comprehensive approach for the choice of motor and transmission in mechatronic applications: Torque peak of the motor. Machines. 2021;9(8):159. doi: 10.3390/machines9080159

[31] Deb K. Multi-objective optimization. Search methodologies. Search Methodol. 2014;2014:403–449. doi: 10.1007/978-1-4614-6940-7_15

[32] Deb K, Pratap A, Agarwal S, Meyarivan T. A fast and elitist multiobjective genetic algorithm: NSGA-II. IEEE transactions on evolutionary computation. 2002;6(2):182–197. doi: 10.1109/4235.996017

[33] Teo TT, Logenthiran T, Woo WL, Abidi K, John T, Wade NS, et al. Optimization of fuzzy energy-management system for grid-connected microgrid using NSGA-II. IEEE transactions on cybernetics. 2020;51(11):5375–5386. doi: 10.1109/TCYB.2020.3031109 33175691

[34] Wang YJ, Wang GG, Tian FM, Gong DW, Pedrycz W. Solving energy-efficient fuzzy hybrid flow-shop scheduling problem at a variable machine speed using an extended NSGA-II. Engineering Applications of Artificial Intelligence. 2023;121:105977. doi: 10.1016/j.engappai.2023.105977

