# Supplementary material for: Selection/control concurrent optimization of BLDC motors for industrial robots
Source: PLoS One. 2023 Aug 16;18(8):e0289717. doi: 10.1371/journal.pone.0289717 (PMC10431662; doi:10.1371/journal.pone.0289717)
Supplement: S1 Appendix — (PDF) [file pone.0289717.s001.pdf]

List of candidate motors. Operation limits

Proposed *off-the-shelf* motors are shown in Tables 5 and 6.

Table 5. List of candidate motors. Operation Limits.

Motor index	Brand	Model	$T_{M,N}$ $[Nm]$	$T_{M,max}$ $[Nm]$	$\omega_{M,max}$ $[rpm]$	I_N $[A]$	I_{max} $[A]$	Suited for joint
1	Maxon	EC60	0.768	11.8	7000	9.56	139	1
2	Maxon	EC60	0.843	6.82	7000	5.9	46.6	1
3	Parker	MPM721	0.660	2	6800	2	6	1
4	Parker	MPM722	1.28	3.8	7000	3.8	12	1
5	Moog	BN3455	2.0267	10.2	4500	23.3	46.6	1
6	Moog	BN3455	2.16	10.2	4500	16.5	33	1
7	Moog	BN3455	2.18	10.2	4500	8.2	16.4	1
8	Moog	BN3425	0.586	2.302	7800	16.4	32.8	1,2,3
9	Moog	BN3425	0.656	2.302	7800	8.7	17.4	1,2,3
10	Moog	BN3425	0.656	2.302	7800	4.4	8.8	1,2,3
11	Moog	BN3435	0.93	3.99	6800	18.74	37.48	1,2,3
12	Moog	BN3435	1.12	4.921	6800	11.5	23	1,2,3
13	Moog	BN3435	1.12	4.921	6800	5.80	11.60	1,2,3
14	Moog	BN3445	1.55	7.55	5500	23	46	1,2,3
15	Moog	BN3445	1.58	7.55	5500	13.7	27.4	1,2,3
16	Moog	BN3445	1.63	7.55	5500	7	14	1,2,3
17	Intecno	BL070480	0.7	2.1	3700	6.5	20	1,2,3
18	Intecno	BL210480	2.1	6.3	3700	18.7	56	1,2,3
19	Samsung	CSM02B	0.640	1.91	3700	1.4	4.2	1,2,3
20	Samsung	CSM04B	1.27	3.82	3700	2.7	8.1	1,2,3
21	Samsung	CSMP01B	0.319	0.950	5000	0.9	2.4	1,2,3
22	Samsung	CSMP02B	0.637	1.91	5000	1.7	4.8	1,2,3
23	Samsung	CSMP04B	1.27	3.82	5000	3.2	9.3	1,2,3
24	Maxon	EC45	0.174	1.38	15000	16.2	119	4,5
25	Maxon	EC45	0.186	0.872	15000	9.65	43.6	4,5
26	Maxon	EC45	0.171	1.54	15000	11.2	93.3	4,5
27	Maxon	EC45	0.184	0.931	15000	6.72	32.6	4,5
28	Maxon	EC45	0.169	1.6	15000	8.55	74.8	4,5
29	Maxon	EC45	0.179	1.56	15000	5.29	43.1	4,5
30	Maxon	EC45	0.191	0.911	15000	3.14	14.5	4,5
31	Maxon	EC45	0.174	1.65	15000	4.21	37.2	4,5
32	Maxon	EC45	0.187	0.962	15000	2.52	12.5	4,5
33	Parker	MPE401	0.15	0.35	5000	0.6	1.7	4,5
34	Parker	MPE402	0.31	0.71	5000	1.2	3.6	4,5
35	Samsung	CSMA3B	0.095	0.29	5000	0.3	0.9	4,5
36	Samsung	CSM A5B	0.16	0.48	5000	0.5	1.5	4,5
37	Samsung	CSM A01B	0.32	0.95	5000	0.9	2.7	4,5

Table 6. List of candidate motors. Parameters.

Motor index	J_m $[\mu Kg \cdot m^2]$	$base/height,depth$ $size[m,m]$	d_m $[m]$	m_m $[Kg]$	L $[mH]$	R_s $[\Omega]$	$Poles$ $[-]$	K_e $[V/rad/s]$
1	83.1	[0.06, 0.17]	12	2.45	0.136	0.172	2	0.084
2	83.1	[0.06, 0.17]	12	2.45	0.41	0.515	2	0.146
3	17	[0.072, 0.16]	11	1.59	8.5	5.5	6	0.427
4	31	[0.072, 0.18]	11	2	3.65	1.85	6	0.427
5	169.37	[0.0826, 0.16]	12	3.266	0.271	0.086	8	0.087
6	169.37	[0.0826, 0.16]	12	3.266	0.482	0.135	8	0.117
7	169.37	[0.0826, 0.16]	12	3.266	1.93	0.504	8	0.234
8	42.34	[0.0826, 0.083]	12	1.050	0.129	0.069	8	0.029
9	42.34	[0.0826, 0.083]	12	1.050	0.575	0.251	8	0.062
10	42.34	[0.0826, 0.083]	12	1.050	2.180	0.941	8	0.121
11	84.68	[0.0826, 0.1]	12	1.760	0.143	0.057	8	0.037
12	84.68	[0.0826, 0.1]	12	1.760	0.432	0.160	8	0.077
13	84.68	[0.0826, 0.1]	12	1.760	1.57	0.575	8	0.148
14	127.03	[0.0826, 0.13]	12	2.499	0.2	0.069	8	0.065
15	127.03	[0.0826, 0.13]	12	2.499	0.45	0.147	8	0.097
16	127.03	[0.0826, 0.13]	12	2.499	1.8	0.552	8	0.195
17	0.08	[0.0023, 0.086]	11	2.1	0.5	0.17	8	0.148
18	0.24	[0.0023, 0.139]	11	4.2	0.155	0.057	8	0.156
19	29	[0.06, 0.12]	12	1.1	9	2.47	8	0.277
20	45	[0.06, 0.14]	12	1.6	3.1	1.07	8	0.280
21	10.787	[0.06, 0.082]	12	0.8	41.2	14.4	8	0.713
22	44.129	[0.08, 0.138]	12	1.8	20	4	8	0.708
23	74.53	[0.08, 0.155]	12	3.1	9.9	1.6	8	0.735
24	11.9	[0.045, 0.135]	8	0.85	0.013	0.050	2	0.011
25	11.9	[0.045, 0.135]	8	0.85	0.039	0.137	2	0.019
26	11.9	[0.045, 0.135]	8	0.85	0.027	0.096	2	0.016
27	11.9	[0.045, 0.135]	8	0.85	0.081	0.276	2	0.028
28	11.9	[0.045, 0.135]	8	0.85	0.045	0.160	2	0.021
29	11.9	[0.045, 0.135]	8	0.85	0.131	0.418	2	0.036
30	11.9	[0.045, 0.135]	8	0.85	0.394	1.24	2	0.062
31	11.9	[0.045, 0.135]	8	0.85	0.197	0.645	2	0.044
32	11.9	[0.045, 0.135]	8	0.85	0.595	1.925	2	0.397
33	2.3	[0.04, 0.072]	8	0.4	12.777	13.015	8	0.28
34	4.1	[0.04, 0.098]	8	0.6	5.952	4.965	8	0.275
35	21	[0.04, 0.119]	8	0.3	40	45.3	8	0.211
36	23	[0.04, 0.119]	8	0.4	21	18	8	0.209
37	43	[0.04, 0.119]	8	0.5	11.3	7.6	8	0.219
